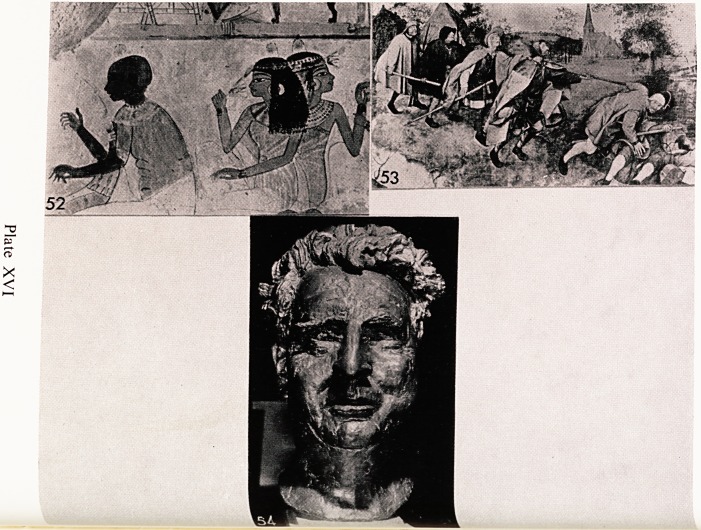# Pictorial Art in Medicine
*Presidential Address to the Bristol Medico-Chirurgical Society, 13th October 1965


**Published:** 1967-04

**Authors:** G. M. Fitzgibbon


### PICTORIAL ART IN MEDICINE *

G. M. FITZGIBBON, M.D., F.R.C.S., J.P.

By custom in the last few decades, the President's address has usually been non-clinical, but loosely connected with medicine or science. He has had free choice of subject, by tradition the title is not disclosed beforehand, and no questions are asked afterwards. All this gives him considerable advantage, and I have therefore chosen a subject which I think is of interest to many people, that of the pictorial aspects of the medical records of history.

It is interesting to reflect on the reasons which prompt human beings to paint a picture, or carve a statue. The commonest and most obvious reason is of course because they want to, and have the urge, which is almost universal, to create something; and I think it is true to say that everyone obtains a gratifying sense of achievement, when he has made or painted something—even although it may only be the garden gate or the front door. Also, painting or drawing may be a very good way of spreading knowledge among members of a society who cannot read or write, and no doubt in the past some artistic works were created solely for this reason.

Paintings are not only a record of events and colourful happenings, but in many instances they give a useful insight into the social and economic background of the times, and are valuable corroboration of other evidence on these aspects of history. In some instances, pictures were painted by great artists, whose fancy was taken by the picturesque quality of many medical and surgical procedures, and sometimes a painter picked out some special feature of current medical gossip as the theme for his picture.

Back in earliest times, medicine was entirely in the hands of priests who practised magic in one form or another. In the long Egyptian civilisation under the Pharoahs this was the case, and the priests were virtually the only people who could read or write. They left a lot of writing and many drawings, and obviously an extensive practice of medicine and surgery went on.

In 450 B.C. Herodotus the Greek historian, when giving an account of Egyptian Medicine, made the following statement :—"Medicine is practised among the Egyptians on a plan of separation, each physician treats a simple disorder and no more. Thus the country swarms with Medical Practitioners, some undertaking to cure diseases of the eye, others of the head, others again of the teeth, others of intestines, and some those which are not local". Specialisation would seem indeed to have started a very long time ago ! Subsequent investigations into Egyptian writings have confirmed his statements and it is known that many specialists did exist as he says, even including one described as "Guardian of the Royal Bowel Movement".

In view of the remarks made by Herodotus, I thought it would be interesting to see to what extent the many specialties mentioned by him were represented by drawings, paintings or sculptures in succeeding centuries, and as so often happens when one starts to look into a subject in more detail, I found there was a bewildering amount of pictorial material which has come down to us today.

* Presidential Address to the Bristol Medico-Chirurgical Society, 13th October 1965

It would be orderly, I suppose, to start with the preclinical subjects. In this connection the main pictorial examples which have survived are mainly anatomical, but there is little in the way of anatomical illustrations until the Renaissance, when the general upsurge in knowledge and skills and arts of all kinds which swept over Europe resulted, among other things, in the development of printing. Illustrated anatomical works began to be produced in the 15th Century, but probably the great turning point in the study of human anatomy came when in 1543 Vesalius produced his first great work. The illustrations were done by well known contemporary artists and all the woodcuts were works of art (Plate VII, 1). The landscape in this well known illustration is part of a continuous landscape panorama, forming the background of the fourteen such muscle figures. Each picture shows a stripping off of muscle layers down to the bare skeleton. During the 1930's the actual panorama was identified, and it is now known that it represents an area of hills to the south-west of Padua.

Rembrandt was interested in anatomy and painted two pictures which have survived. One is Dr. Deeman's anatomy lesson, and he can be seen holding the vault of the skull in his left hand (Plate VII, 2). The other anatomical picture (Plate VII, 3) is better known and he was only 27 when in 1632 he was commissioned to paint the anatomy lecture of Dr. Nicholas Tulp, and the Royal College of Surgeons have a copy of it. Tulp was Professor of Anatomy in Amsterdam, and the picture was commissioned for the Corporation of Surgeons and hung in their anatomy theatre. The corpse was that of a malefactor and his body was dissected on January 31st, 1632. The picture was bought for the National Gallery at the Hague and £3,500 was paid for it in 1828. It must be worth an astonishing sum today.

One aspect of Rembrandt's work was the almost photographic accuracy of it, and it is therefore all the more surprising that he should have made the grave anatomical mistake which you can see in this picture. He has put the forearm muscles of the right forearm on to the left side.

Rembrandt, like many artists, was chronically hard up and quite irresponsible in money matters. He was unable at times to afford to employ models, and used his mother (Plate VII, 4) on a number of occasions. This portrait of her shows what I mean about the photographic accuracy of his work, because if you look at the side of her nose near the left eye you can see a typical basal cell carcinoma or common rodent ulcer which he has faithfully recorded.

A later Professor of Anatomy at Amsterdam, Willem Roell, was painted in his dissecting room by Cornelius Troost in 1728. In this colourful picture, he is seen demonstrating some points in connection with the knee joint (Plate VII, 5).

Thomas Rowlandson, the great English caricaturist, has put on record a scene (Plate VIII, 6) which has been identified as William Hunter's dissecting room. It was an attic, and two skeletons are seen on the left and a third one in the background. Two bodies are being dissected and a third is lying on the floor to the left. He also couldn't resist doing a charming caricature of two grave-robbers who are shown putting the shrouded corpse into a sack (Plate VIII, 7). A skeleton leans over them holding a lamp in its right hand. On the coffin lid is inscribed " RESURGAM ". With a nice sense of humour he has entitled the picture " The Resurrectionists ". Both these engravings are among the Royal College of Surgeons' collection.

Plate VII

6

7

9

10

Descriptive system of personality. Inner circle shows the "four temperaments" of Hippocrates and Galen; outer circle shows, patterning of personality traits according to modern research.

8

Plate VIII

Probably the real beginning of Medicine in history was the work of Hippocrates. He is supposed to have been born on the island of Cos off the coast of Asia Minor about 460 B.C. He is spoken of as belonging to a sect—the sons of Asclepios. It is said that on his father's side he was descended from Asclepios, and his early medical studies probably were at the famous Temple and Medical Centre of Asclepios on the island of Cos. Very little is known about him, and he is said to have died in Marissa at an advanced age.

A mass of works known as the Hippocratic Collection has been handed down. It is not known how many, if indeed any, of them are genuine. Some go back to 5th Century B.C. but they have been copied and modified and added to, and the earliest known copy is about 7th Century.

There are several surgical works and they are astonishingly modern in some of their statements and include details like this :—" the nails of the operator neither to exceed nor fall short of the finger tip. Practise using the finger ends. Practise all the operations with each hand, and with both together, your object being to attain grace, ability, speed, painlessness and readiness. Let those who look after the patient, present the part for operation as you want it, and hold fast the rest of the body so as to be all steady, keeping silence, and obeying their superior ".

Hippocrates sorted people out into four well-known groups, melancholic, choleric, sanguine and phlegmatic (Plate VIII, 8). It is possible to elaborate these four main groups as this diagram shows, and Eysenck quotes several authorities for the statement that there is a direct relationship between cancer and extroversion and stability. One source he quotes states that the death rate among stable types from lung cancer was 256 per 100,000 and that of the unstable was 56 per 100,000. One wonders whether Hippocrates' simple classification of people may have a much more profound and significant meaning than has hitherto been thought?

I mentioned that Hippocrates is referred to as belonging to a sect—the sons of Asclepios. It is probable that he received some or all of his medical training from the large medical centre which existed at that time on the island of Cos. Aesculpaius in Latin or Asclepios in Greek was " the Greek God of Medicine —the Son of Apollo ". He is said to have come from Thessaly and historically he had a great reputation as a skilful physician, and temples and buildings were erected in many parts of Greece.

An extensive cult of temple healing grew up and lasted some hundreds of years. These centres were more than religious temples and assumed the character of what we would call sanatoria. The largest and best known was that at Epidauros where there was a vast complex of buildings from about 4th Century B.C. It was magnificently situated on a plain close to a hill and surrounded by woods. The hill served to provide the theatre—one of the finest ever built in Greece (Plate VIII, 9). It has a seating capacity for 14,000 people. The complex of buildings consisted of gymnasia, baths and exercise quadrangles and they formed a quiet refuge in pleasant surroundings where sick, tired and worn out people could recuperate. The theatre and other recreations were a big feature to encourage relaxation and distract the sick from their problems. A great deal of the treatment that went on was carried out under the influence of drugs, and in drugged sleep the God appeared, and spoke and advised them about their problems. As these had previously been discussed with the priests the God was well informed about their troubles. Considerable use was made of baths, exercise, diet, medication, plays in the theatre, and in addition manifestations and advice during what seemed to the patient to be a dream. No matter what the technique was, there are plenty of records on tablets left by patients, that they had seen the God in a vision, and that he had cured them. The theatre is largely intact today and is well worth seeing. Unfortunately little remains now of the complex of the main buildings.

In some ways medicine does not lend itself to illustration as well perhaps as surgery or the specialties, but there are many interesting works of art which put on record some aspects of the physician's work. From an early date in recorded history, dwarfs have provided a subject for sculptors and artists, and they have formed part of the staff of the courts of kings and princes, and in the middle ages many of the courts of Europe had a number of dwarfs on the staff right up to the time of the French Revolution. It is on record that a Cardinal in Rome managed to get hold of 34, all with ugly faces, to serve at a banquet! A delightful group is of the court dwarf Seneb, and his family (Plate VIII, 10). It is about 12 inches high, and was found in the Necropolis at Giza. The Egyptians believed that these statuettes could replace the body if it decayed and so provide a home for the dead man's spirit. In this statuette, Seneb is clearly an achondroplasiac dwarf, and the artist, in an effort to add to Seneb's dignity and lessen the effect of his deformity, shows him seated on a pedestal. This, in turn, is partly concealed by his two normal children who stand in front of it. By convention in Egyptian art, children were indicated by the gesture of the finger in the mouth. Also by convention children under puberty were shown naked, and males were indicated by the sidelock of hair. The date of this work is about 2,500 B.C.

Dwarfs formed part of the staff of many courts, and the famous Spanish artist Velasquez painted a number of them when he was the King's personal artist, and was attached to the court. One of his first pictures of dwarfs (*circa* 1650) was an achondroplasiac called Sebastian de Morra (Plate IX, 11). He is shown sitting on a table, very elaborately dressed and facing the viewer. He has one of the most wonderful heads ever painted by this artist.

Plate IX, 12 is a portrait of one of Philip IV's Court Jesters and is called Don Antonio—The Englishman. He was obviously one of the more important dwarf court jesters, judging by his very fine clothes. He is not an achondroplasiac but is a perfectly proportioned miniature due to absence of growth hormone.

One of Velasquez's best known works is entitled "The Maids of Honour" (Plate IX, 13). It shows the little Princess Margarita in the centre and a female achondroplasiac dwarf attendant on the right of the picture. Velasquez himself can be seen on the left. He was painting the King and Queen who can be seen in the mirror at the back. The general style, with the use which is made of the mirror and the open door in the background, is reminiscent of the Dutch or Flemish School.

Plate IX, 14 in the National Gallery and is usually known as the Ugly Duchess. It must surely represent a pituitary disorder? Her features look in some ways acromegalic. The picture is attributed to Quentin Massys and is thought to have been painted about 1500 A.D. Whilst on the subject of pituitary dysfunction, a portrait of a man called Daniel Lambert is in the Royal College of Surgeons' Collection (Plate IX, 15). It is stated that he weighed 52 stone. He was born in 1770 and not surprisingly didn't live very long; he was 39 when he died.

With the awakening of scientific curiosity in the Renaissance, attention was directed to the urine, and this fluid came to have a seriously exaggerated importance. Physicians noted the smallest variations in colour, odour, and sediment (Plate IX, 16). Significance was attached to the changes which occur after standing exposed to the air for several days. No less than 60 different shades were recorded, and many different odours. The urine flask was shown so often in the hands of physicians, that it almost became the symbol of the profession. Uroscopy as it was called is said to have arisen as a result of ecclesiastical restrictions on priests and monks who were not allowed to visit patients, and so the practice started of making diagnoses, and advising treatment, from looking at the urine. Incidentally some doctors were not above reproach in this matter and there is an old statute of the Royal College of Physicians in which members were forbidden to give advice upon mere inspection of the urine.

Many artists have painted pictures of the visits of physicians. In the picture by Jan Steen (Plate X, 17) the girl has fainted, and her mother has been burning something in the bowl which is on the floor, the fumes from which were supposed to help in bringing her round. Steen has used the fairly common Dutch technique of an open door in the background to help in adding interest and depth to the picture.

A nice example of a sick patient is a self portrait by Goya (Plate X, 18). It shows him desperately ill in the arms of his doctor. He did recover, but died about two years later. He gave the picture to his doctor as a token of his gratitude for the care he had taken of him.

An expression of face which was seen not infrequently in my earlier life can be seen in the study by Adrien Brouwer entitled " Nasty Medicine " (Plate X, 19). In this age of pills, tablets, and injections such expressions are hardly ever seen.

Surgery lends itself better in some ways as a subject for artists. The procedures often have a dramatic quality which catches their imagination. An illustration from a 15th Century manuscript looks quite exciting and dramatic (Plate X, 20). It shows a patient being bled, and it appears very much as if the doctor has opened the bracial artery judging by the way the blood appears to be spurting from the arm !

Some surgeons had a sign-board, and there is a replica of one in the Royal College of Surgeons (Plate X, 21). The original was found at Poole in Dorset but unfortunately it was almost completely destroyed when the college was bombed in May 1941, and only fragments remain. This surgeon is seen in the centre; in the compartment above he is shown examining a flask of urine, to the left from above down can be seen venesection, amputation of leg, and extraction of teeth, and to the right reduction of dislocated shoulder, examination of a tumour, and testing the action of the back muscles. It bears the date " Anno Domini 1623 " and the inscription reads " The Almighty provided remedies out of the earth and the wise man will not despise them ".

When reading about surgery in the middle ages and looking at the diagrams, one cannot help but be amazed at the superhuman endurance of the patients. Venereal disease and uncontrolled infections of all kinds were rife, and infections of the urogenital tract must have been almost universal. This led inevitably to stone formation on a scale which we cannot imagine today. It is amazing to think that patients were tied up in the lithotomy position or with their feet tied to their knecks, often the hands and bodies tied down as well, while a surgeon operated for a stone (Plate X, 22). This illustration is from a manuscript dated about 1510. Two fingers were inserted into the rectum, the stone was worked down toward the neck of the bladder and then extracted through an incision made in the perineum. Both patients and surgeons must have been very tough!

General practice in those days necessitated some surgery on the daily round. Plate XI, 23 is a painting of such an event by Brouwer. It is almost certainly a self-portrait and he has put his own face on the patient's body. Brouwer was a man who lived among the poor and shared their life. He painted many scenes of life in pubs and low dens, and he depicted the poor under-nourished stunted peasants wearing torn clothes and living in awful conditions of dirt and misery. They tried to overcome this by drink and tobacco, both of which were cheap. The latter was often blended with some narcotic and his pictures frequently show smokers overcome by their addiction. In Plate XI, 24, the woman has passed out completely and her pipe is shown in pieces on the floor. Plate XI, 25 is another general practice scene and it shows the village doctor operating on a patient's foot. Behind him stands a woman with a reserve knife, and she is turning her head to look at the intruder at the door. In the background the doctor's trainee is examining another patient in a rather nice barrel chair.

All through history, orthopædic conditions of one sort or another have been painted and illustrated. Plate XI, 26 is a beautiful example of incised carving which was painted about 1500 B.C. It is a wall painting from an Egyptian tomb and shows a man who has a severe talipes equinus. The calf of the right leg is grossly wasted, and almost certainly it is an example of poliomyelitis affecting the calf muscles.

In a Florentine manuscript of the 9th Century there is a commentary with illustrations from the work of Appolonius of the first century B.C. It contains a very nice illustration of a Greek surgeon reducing a dislocated jaw (Plate XI, 27). Quite correctly he has his hand in the patient's mouth pressing downwards and backwards on the molar teeth.

From the time of Hippocrates to the present day—perhaps one ought to say especially at the present day—the human back has caused trouble. Dislocations, prolapsed discs, displacements, malalignments have all caused pain, disability and even death. A basic principle in the treatment of many back conditions is traction, and Plate XII, 29 taken from a 10th Century Byzantine manuscript, shows a method used in the first century B.C. by Greek surgeons. You will readily recognise that the surgeon and assistant are the same characters as were shown to be reducing a dislocated mandible in the previous picture. The text says that after binding the patient to the ladder, the latter should be raised against a high tower or a house and the ground below should be very firm. It goes on to say that it is a very skilled procedure and the two operators must work exactly together so that the two ends of the ladder strike the ground at precisely the same moment.

Continuing with the subject of backs, I feel I must show you this wonderful piece of machinery (Plate XII, 29). It is the Scamnum as used in Hippocratic times. A perfect 16th Century model was found in central Italy in 1924. Without going into details you can see the principle. It was used in Hippocratic times not only for back injuries, but also for reducing dislocated hips which frequently occurred as a result of wrestling contests. It was also used to straighten spinal curvatures. Talking of spinal curvatures Plate XII, 30 is a photograph of a very nice little statuette of the Early Aztec period of South America. It undoubtedly shows an angular kyphosis and suggests that this race experienced one at least of the manifestations of tuberculosis.

Owing to the inability to control infection, amputations were commonplace and of course undertaken without anæsthetics. Patients were fiitted with artificial limbs of a sort. Plate XII, 31 is a sketch of a man with an artificial leg—it is in fact a sketch of the Marquis of Anglesey who lost his leg at Waterloo. His leg was subsequently amputated and the account reads as follows : He never moved or complained, no one even held his hand. He said once quite calmly that he thought the instrument wasn't very sharp. When it was over, he did not appear in the least shaken and the surgeon said his pulse hadn't altered. He is shown in this sketch some years later talking to the Duke of Wellington about old times.

The dentists have been freely represented by artists in the past. Hygiene in the past was I fancy less good than today and toothache, alveolar abscesses, and gum boils must have been more common. Some of you who know Wells Cathedral may recognise the carving in Plate XII, 32. It is on one of the capitals in the south transept and this man has for a good many centuries been trying to draw attention to his aching tooth. In the villages, the local smith was often the dentist and in a picture by Hans Ludwig Smidth (Plate XII, 33) there seems to be a certain amount of tenseness. The smith, tongs in hands, is rolling up his sleeves and the patient is looking at him rather anxiously; her mother is holding her arm and obviously offering encouragement. In a picture by Schleisner (1810-1882) the tables seem to be turned on the smith who is the patient (Plate XIII, 34). The doctor (who has some instruments in his pockets) is obviously concentrating very hard on what he is doing and the smith looks absolutely scared stiff and the whites of his eyes are showing.

For some curious reason quite a number of medical and dental procedures seem to have been performed in public, and an audience inevitably gathered round which must have been embarrassing for both patient and dentist. Jan Steen has portrayed the unfortunate boy in Plate XIII, 35 at an awkward moment, when judging by his expression and movements, he seems to be in great discomfort. The picture is entitled "The Tooth Master" and it is full of interesting detail.

Among the many things throughout history which have been credited with magic powers, teeth have from time to time been popular. A grotesque drawing in a special aquatint technique was done by Goya in one of his psychotic attacks (Plate XIII, 36). Teeth taken from a person who had been hanged were supposed to have special magical powers, and Goya shows this woman trying to extract teeth from a corpse hanging on a gallows. She is obviously loathing her job and her left hand is holding a cloth up between her face and that of the corpse. Goya mounted a vehement attack against injustice, false pride, clericalism, drunkenness, and in this drawing he is attacking superstition.

Neurosurgeons were active in early primitive times and many skulls have been found with holes trephined in them. This was done for fits, headaches, epilepsy, and madness; the idea being to let the devil out. Many of the victims survived as can be deduced from the holes in the bone which show signs of repair.

In a photograph of an Aztec jug (Plate XIII, 37), the handle is the figure of a surgeon turning back a scalp flap, with an instrument in his right hand.

Brueghel painted a picture of an operation on the head which looks to me very much as if the surgeon is about to remove a sebaceous cyst. The unfortunate patient seems to be very well secured in the chair (Plate XIII, 38). Another painting in the Royal College of Surgeons' Collection shows a surgeon operating on a patient's head (Plate XIII, 39). The artist is not known, but was probably Dutch. It is interesting to see how little attention the surgeon seems to be giving to his work!

Jan Steen, who was very interested in medical subjects, recorded a scalp operation (Plate XIV, 40). This picture is called " The Quack ". A lucrative racket in those days was to go round from village to village and offer to do curative operations for fits, headaches, and insanity. The surgeon made a hole in the scalp, and then by sleight of hand he produced a bloody stone from somewhere, which he exhibited in triumph, and if he had done his trick well, the audience supposed it had come out of the unfortunate patient's head. In this picture the victim can be seen in the centre tied to a bench, the surgeon stands behind holding up the bloody stone for all to see. There are a number of onlookers, and in the front a woman appears to be wheeling the previous patient away! The quack having been paid leaves the village quickly, and goes on his way before the victim has had time to have another fit.

Maxillo-facial and plastic surgery has been the theme for a good many drawings and paintings. Reconstructive surgery of the nose was practised in India before Christianity. Cutting off the nose was done as a penalty for certain crimes, it was also done for adultery, and a certain number of noses were also lost in wars. The Indians developed a method of repairing the nose using a forehead flap. It seems extraordinary that they should have had any success, but clearly they must have had, as the work continued. In a periodical published in the year 1794 was a communication from a correspondent in India which was accompanied by a portrait of the person mentioned (Plate XIV, 41). Cowesjee was a bullock driver for the British Army in 1792 and was captured by Tippo who cut off his nose and one of his hands. A Maharatta surgeon near Poona put on a new nose. The periodical also gave an eye witness account by two medical gentlemen who said they had seen one of these operations done. In 1816 an English surgeon, J. C. Carpue, published a book in which he described the Indian and Italian methods of rhinoplasty, together with a description of a case of his own. It seems to have gone well, and Plate XIV, 42 is an illustration taken from his book. The Italian method to which he refers was that of Taliacotius, who was appointed to the chair of Anatomy at Bologna about 1586. His method of rhinoplasty was to use a direct flap from the arm, and the diagram (Plate XIV, 43) is of course extremely well known. Occasionally this method is still used today. He must have had a fair measure of success as he flourished exceedingly and wrote a book in 1597 describing the principles for reconstruction of noses, lips and ears. He used his house as a nursing home and had to enlarge it. It is said

Plate IX

Plate X

Plate XI

Plate XII

28

29

30

31

32

33

Plate XIII

Plate XIV

Plate XV

46

47

48

49

50

MASK FOR HEALING
51 CROSSED EYES, 1583

53

52

54

Plate XVI

that on one occasion he had in it 12 German Counts, 19 French Marquesses, 100 Spanish Cavaliers and 1 English Nobleman. At one time in his career he took out a patent and so obtained a monopoly. His fees varied according to the type of nose he provided—High Roman noses cost most.

I mentioned earlier that artists were sometimes inspired by current medical gossip, and about the year 1490 Fra Angelico painted a picture (Plate XIV, 44) a miracle performed by Saints Cosmos and Damian, who were well known for their good works and wonderful cures. They were martyred by Diocletian in A.D. 303. The story is that a Deacon named Justinian suffered from a malignant condition of his leg, and he went to pray in a church in Rome. After a time he fell asleep, and dreamt that the two Saints stood beside his bed. The saints having decided to amputate the leg recalled that a Moor had just been buried in the cemetery attached to the Basilica of St. Peter. They departed and later returned with the appropriate leg from the dead Moor and grafted it on to Justinian. I have seen paintings of three other versions of this occurrence and Plate XIV, 45 is one of them. The patient in this version was a soldier whose leg had been very badly injured. The Saints have been very tidy and have disposed of the amputated white leg by putting it on the body of the Moor. The idea of grafting a limb was there, but they were many hundreds of years ahead of their time, because the first account of successful replacement of an amputated limb appeared in the Lancet in May, 1964, written by R. J. Horn. It was, of course, the replacement of the patient's own limb, and not a homograft as illustrated in these paintings.

A charming and well known picture by Ghirlandhaio (Plate XV, 46) shows the old man to be the possessor of a nose which exhibits a moderate degree of rhinophyma. This condition turns up from time to time and I suppose we operate on two or three each year.

Coming up to more modern times the Royal College of Surgeons has among its collection a coloured sketch by Tonks of Sir Harold Gillies in the first World War, operating on a soldier who had been wounded in France (Plate XV, 47). Tonks was a Fellow of the College, but was an artist of great ability and didn't practise surgery, and he became Slade Professor of Fine Art in the University of London. He returned to medicine in the war to assist Gillies.

Pædiatric illustrations are not as common, I found, as some other subjects. I did find several, and among them this (Plate XV, 48) particularly striking one. It is by Gabriel Metsu who was one of the most important of the minor Masters of the 17th Century (Dutch). The picture is in the Louvre. The scene is a fairly typical Dutch interior and an anxious woman has a very sick child on her knee, who appears sunken eyed and is lying back in her mother's arms in a very limp way. I wouldn't like to have to guess at the diagnosis—it could be one of several conditions.

The Royal College of Surgeons' collection also has this portrait of an albino child (Plate XV, 49). She has pink eyes but I am afraid they don't show up very well. The picture is inscribed in the lower left hand corner MacDonnel aged 1 year and 8 months, born in Spitalfields in 1781.

The Royal College also has what must surely be a unique picture of Siamese twins (Plate XV, 50). It is by Irvine and dated 1830. These two boys were born in 1811 near Bangkok. Their father was Chinese and their mother

Siamese. Their names were Chang and Eng, and when born the bridge which connected them was so short that they were face to face, but it stretched as they grew, and they were very active and swam a lot. They travelled widely with various circuses and settled in North Carolina where they married two sisters. They lived with their respective wives and children (Chang having 10 and Eng 9) about a mile and half apart, passing three days alternately at each house. Eng was amiable and sober and Chang was irritable and a heavy drinker. One interesting feature which suggests that their circulations were not connected was the fact that an illness of one had no effect on the other. Eng never had any hang-over from Chang's drinking, and sometimes one would have a temperature while the other was normal. In August 1870 Chang had a stroke from which he never fully recovered and four years later he died. Eng died a few hours afterwards.

Eye conditions and diseases are illustrated fairly often. A number of mediæval manuscripts show a surgeon sitting in front of a patient with a huge knife about to operate on the eye, and operations actually were done for cataract, but with what success I don't know—it seems difficult to believe they had any! I came across an illustration of a fascinating mask which was designed for the purpose of curing squints (Plate XV, 51). The idea was good, and indeed today the Orthoptists do a lot of good work in exercising patients' eye muscles.

The commonest aspect of eye work which has been recorded is blindness. This of course was appallingly common; venereal diseases were prevalent, babies' eyes were infected, and many were blind from birth. It is rather less than 100 years since the Credes method of putting a drop of silver nitrate in the eyes of new born babies brought about an enormous reduction in the number of blind children. One of the earliest illustrations of blindness must surely be a painting from the tomb of a noble near Luxor (Plate XVI, 52). It is of a blind girl playing a harp to a group of ladies. Its date is about 1400 B.C. Blindness has provided artists with subjects for pictures on many occasions, and the pathetic state of columns of blind beggars finding their way around gave Pieter Brueghel the inspiration for the picture (Plate XVI, 53) which he called the " Parable of the Blind ".

In our own Art Gallery here in Bristol we have a magnificent bronze head by Sir Jacob Epstein (Plate XVI, 54). It is, as some of you no doubt recognise, the head of Professor MacInnes, who is well known to many of you. He was blind as an undergraduate, but took part in a variety of activities and never allowed his misfortune to interfere with the things he wanted to do. One who knew him well tells me that in earlier days he saw him " turn a cart-wheel ". He used regularly to row in a four, and also skated. This bronze is a great work of art and it shows well the characteristics of courage, patience and fortitude which enabled him to carve out a distinguished career in this University. In some curious and rather subtle way, it conveys to the onlooker the feeling that this is a representation of a blind man.

I think you will agree that an astonishing range of medical treatment and surgical procedures have been carried out in the past, for the works of art about which I have spoken are part, but only a very small part, of the medical records of history.

*Acknowledgements :*

I am grateful to the Council of the Royal College of Surgeons of England for permission to take photograps of some of their Collection of Pictures, and to make use of Illustrations Nos. 6, 7, 15, 21, 39, 49, and 50.

I gladly acknowledge the help which I have received from Mr. and Mrs. Banham of the Photographic Department, Frenchay Hospital, Bristol.

REFERENCES

*Magic and Medical Science in Ancient Egypt.* Paul Ghahongin. Hodder and Stoughton, London, 1963.

*L'Art et La Medicine au Musée de Colemer.*

*The Dentist in Art.* Pindborg and Marvitz. George Proffer Limited, London.

Edwin Smith. *Surgical Papyrus.* Vol. I Breasted. Chicago Press, 1960.

*Pathology of Infancy and Childhood.* MacGregor Livingston, 1960.

*The Life and Times of Gaspare Tagliacozzi.* Herbert Reichner, New York, 1950.

*Goya.* (The Gallery of Great Masters) Dina Formaggio. Oldbrune Press, London.

*Rembrandt* (Spring Art Books) Trewin Copplestone. Spring Books, London.

*Anatomy of Art No. 102.* Vol. III, No. 1. Nelson Gallery and Atkins Bulletin. Kansas City, Missouri.

The Debt of Medicine to the Fine Arts. Nixon. *Bristol Med. Chir. Journal.* 1922-23. XL, 1-29.

*L'Art et La Medicine.* Paul Richer, Gaultier. Magnier et Cie, Paris.

*Dutch Painting.* R. H. Wilenski.

*Looking at Pictures.* Kenneth Clark, John Murray, London.

## Figures and Tables

**Plate VII f1:**
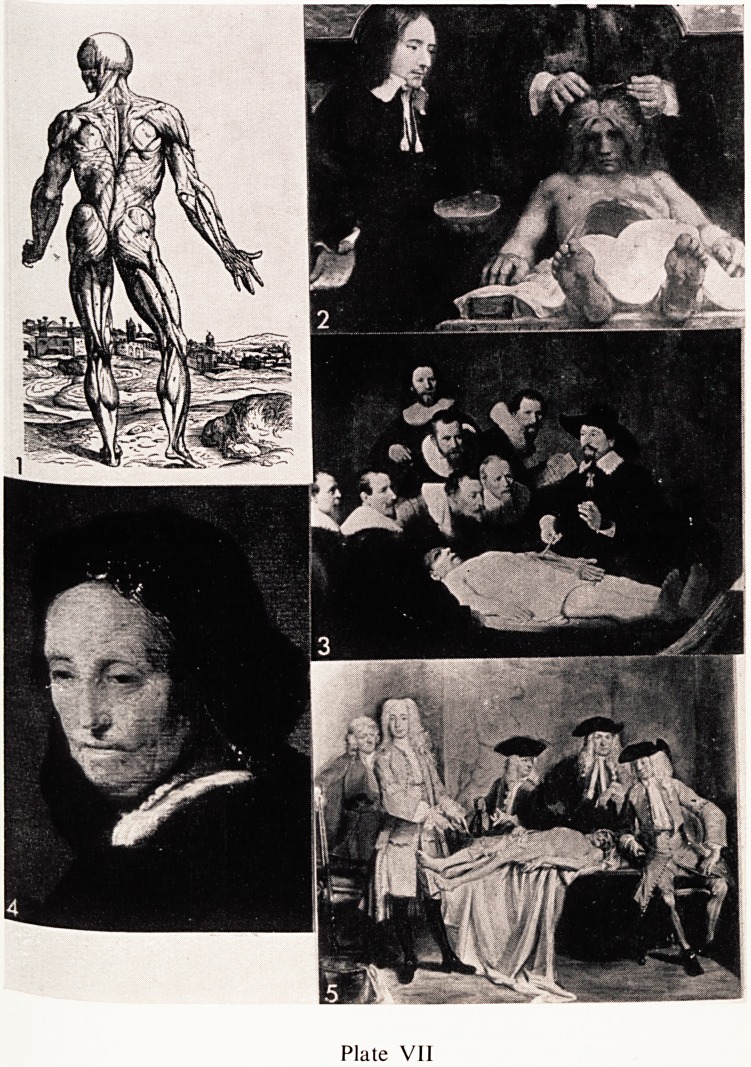


**Plate VIII f2:**
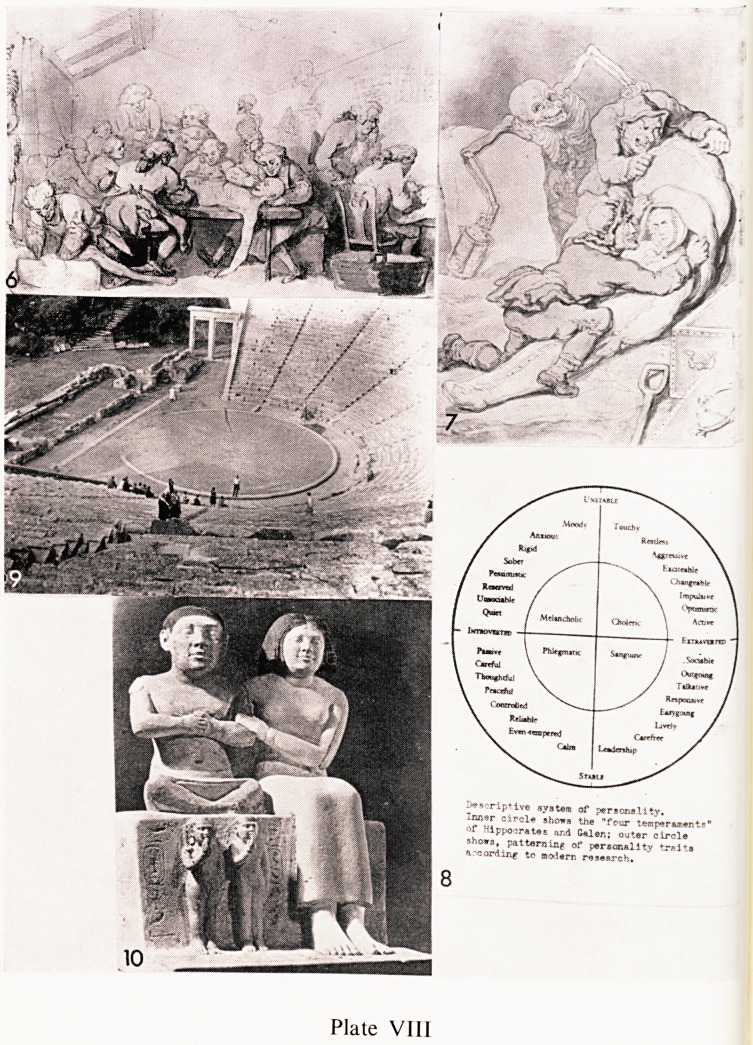


**Plate IX f3:**
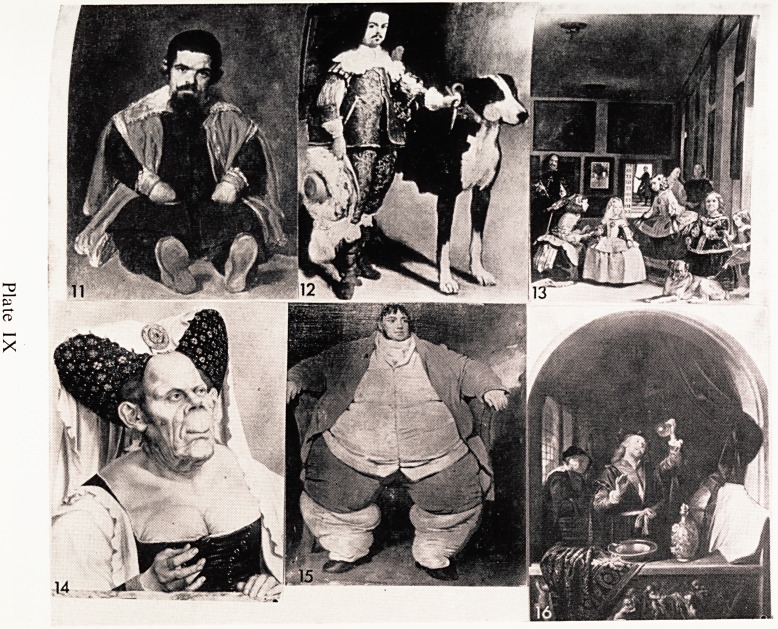


**Plate X f4:**
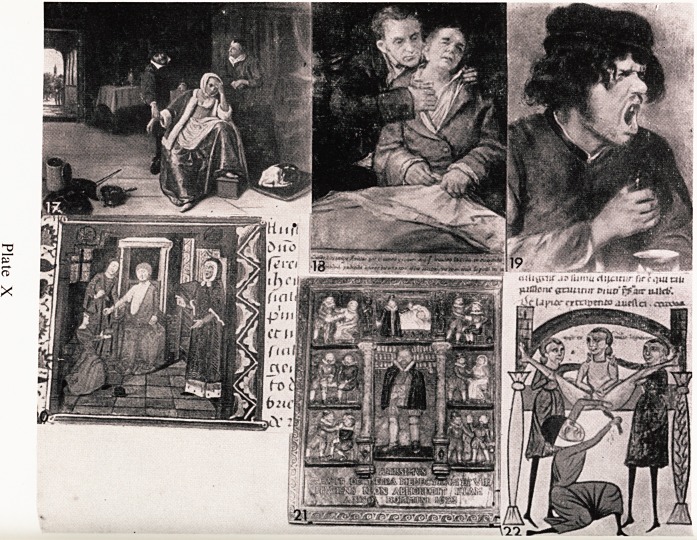


**Plate XI f5:**
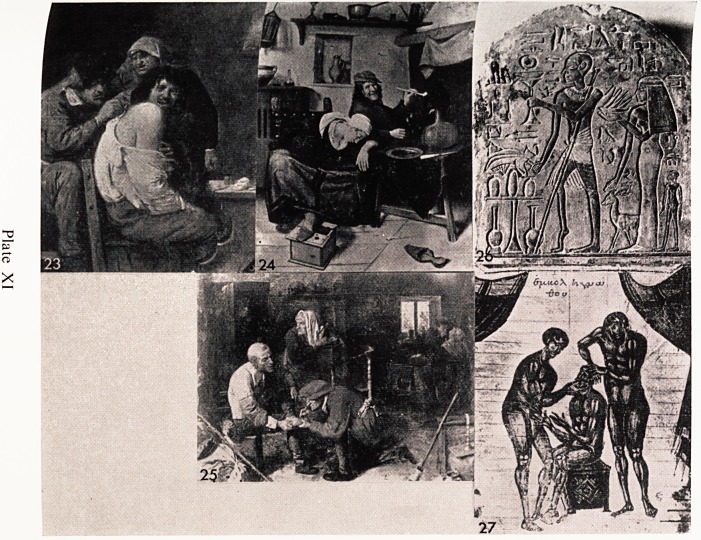


**Plate XII f6:**
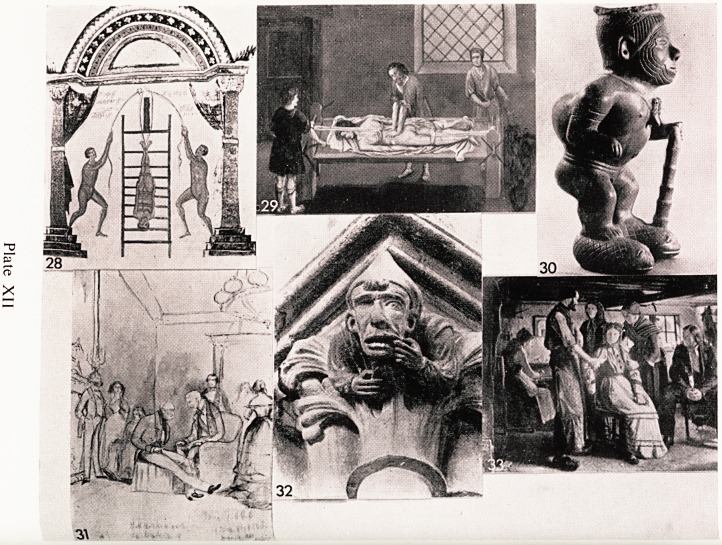


**Plate XIII f7:**
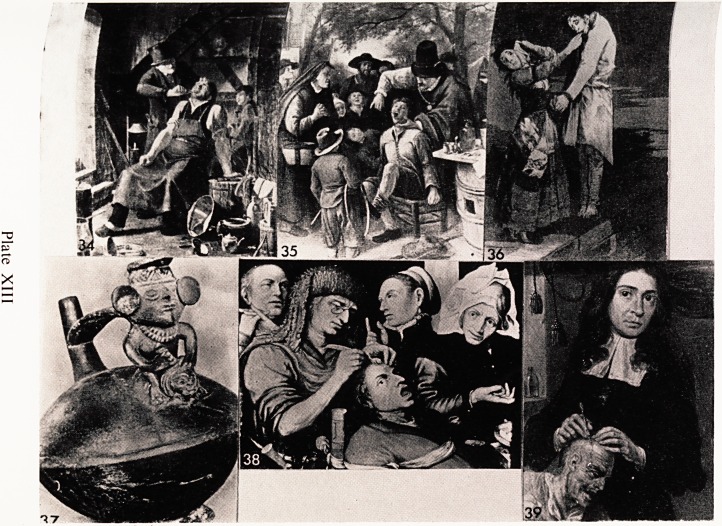


**Plate XIV f8:**
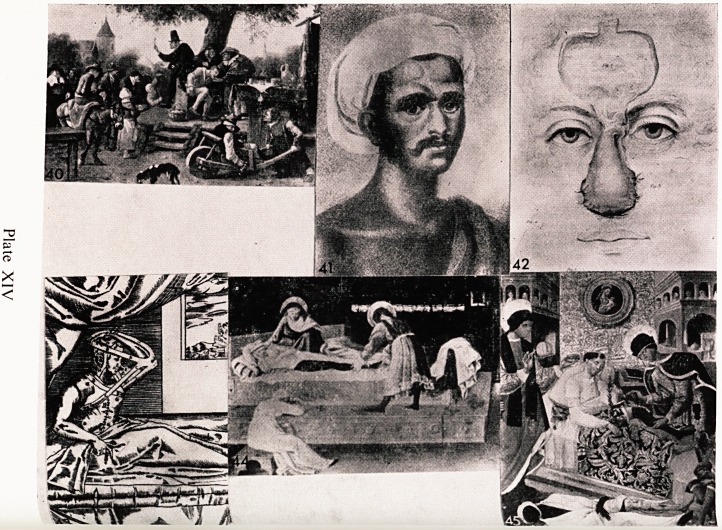


**Plate XV f9:**
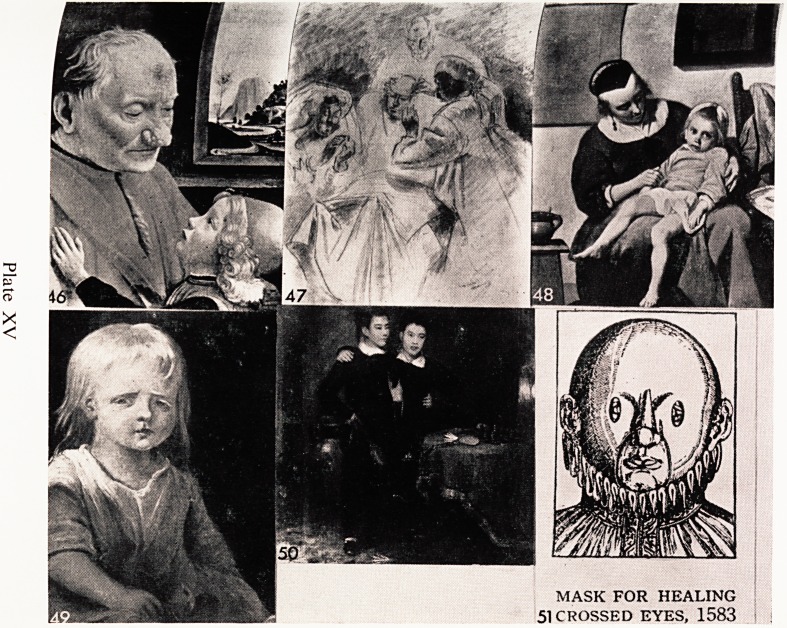


**Plate XVI f10:**